# “That’s Where Our Income Comes From”: Women’s Perceptions of Links Between Reproductive Struggles and Hydraulic Fracturing

**DOI:** 10.3389/fsoc.2021.623222

**Published:** 2021-05-13

**Authors:** Mollie K. Murphy, Mehmet Soyer, Sebahattin Ziyanak, Taya Godfrey

**Affiliations:** ^1^ Department of Languages, Philosophy, and Communication Studies, Utah State University, Logan, UT, United States; ^2^ Department of Sociology, Social Work, and Anthropology, Utah State University, Logan, UT, United States; ^3^ Department of Social Sciences, The University of Texas Permian Basin, Odessa, TX, United States

**Keywords:** hydraulic fracturing, environment, reproduction, pregnancy, economics

## Abstract

Reproductive hardship is highly stigmatized, which leads to such struggles being relegated to the private sphere. At the same time, numerous studies show links between toxic chemicals and reproductive hardship including miscarriage, infertility, and birth defects. There thus exists a disconnection between *structural* contributors to reproductive challenges and the fact that such hardship is frequently viewed as a *personal* problem. Considering this tension, this qualitative study sought to examine how women who had both experienced reproductive difficulty and lived proximal to hydraulic fracturing operations made sense of their experiences. Analysis revealed that participants emphasized hydraulic fracturing as economically essential at the same time that they tended to minimize fracking as a potential contributor to reproductive hardship.

## Introduction

High-volume hydraulic fracturing (fracking) is a relatively new and controversial technique of extracting oil and gas from shale formations deep beneath the earth’s surface. Initially discovered in 1997, the practice combines more traditional methods of extraction with a horizontal drilling technique that involves fracturing shale with a “cocktail” of chemicals, sand, and water (Rinaldi, 2015). Since 2008, a boom in fracking has brought temporary economic prosperity to rural communities at the same time that it has created significant environmental and human health concerns (Fitzgerald et al., 2016; Murphy, 2020). While it is difficult to prove causal links between chemicals used in fracking and health problems, available research illustrates “significant associations” between emissions from fracking and negative health effects (Wollin et al., 2020; see also; Mrdjen and Lee, 2016). Although men make up 85% of the oil and gas extraction industry workforce (Energy Workforce and Technology Council, 2018), women and infants living proximal to fracking operations face unique health risks. Multiple studies have linked proximity to fracking to reproductive hardships including preterm birth (Whitworth et al., 2018), decreased birth rate and decreased health (Currie et al., 2017), and congenital heart defects (McKenzie, Allhouse, and Daniels, 2019). Despite such findings, struggles with reproduction are stigmatized and personalized, which in turn creates difficulties for those seeking to expose the harmful effects of toxic chemicals (Layne, 2001). The fact that mainstream news depicts fracking as either an economic benefit *or* an environmental concern further complicates this problem (Krause and Bucy, 2018). That is, those who frame fracking as an environmental concern are frequently criticized as threatening a community’s economic welfare made possible by the oil and gas industry.

Despite significant concerns about the effects of fracking on reproductive health, the perceptions of women suffering such effects remain underexplored in scholarly research. Accordingly, this qualitative study examined the extent to which women who have experienced significant reproductive hardship and live near fracking operations attribute their struggles to their proximity to fracking. This study is grounded in Cognitive Dissonance Theory (CDT), which captures the ways in which individuals negotiate incongruities between cognitions or between cognitions and behaviors (Harmon-Jones and Mills, 2019). The experience of dissonance leads to psychological discomfort; this, in turn, “motivates the person to reduce the dissonance and leads to avoidance of information likely to increase the dissonance” (Harmon-Jones and Mills, 2019, p. 3). We consider CDT in the context of communities economically dependent upon oil and gas extraction while suffering negative health effects, focusing specifically on the Uintah Basin area of eastern Utah. In Uintah County, mining, quarrying, and oil and gas extraction make up the largest economic industry in the area (Data United States of America, 2018), making environmental and human health concerns related to fracking especially controversial within the community. As chronicled in a 2015 issue of *Rolling Stone*, a midwife living in the Uintah Basin noticed growing rates of stillbirth, miscarriage, and birth defects in the region around 2013. When she questioned whether such problems were linked to fracking operations, she received death threats and challenges to her reputation (Solotaroff, 2015). It is within this context that our research participants grappled with economic dependence on fracking and reproductive challenges.

## Method

After receiving IRB approval, we recruited six women from the Uintah Basin region. One of the researchers with connections to the area posted recruitment materials to Facebook inviting those who met the criteria to contact the researchers. Women were eligible to participate if they self-identified as people who have experienced reproductive challenges (e.g., still birth, birth defects, miscarriage) and lived in the Uintah Basin region. While some participants were recruited via social media, others were recruited via snowball sampling. Interviews concluded with the researcher asking the participant to share our contact information with anyone who met the criteria and was able and willing to participate. In each case, participants made the first contact with the researchers. Participants were asked to take part in semi-structured interviews in exchange for ten-dollar gift cards to Amazon in compensation for their time. Participants were paid regardless of their answers. Women ranged in age from 31 to 42 years old, with one participant not reporting their age. Participants were mothers to two to six children.

Interviews were conducted in late April of 2019. In order to gather an in-depth understanding of the extent to which women who had experienced reproductive challenges connected their struggles to fracking in the area, we asked overarching questions such as, “When did you first become aware of hydraulic fracturing or fracking? Do you believe that fracking poses a threat to public health or safety? To what extent has your experience with pregnancy impacted your perception of fracking?” To analyze the interviews, we employed a thematic analysis using the constant comparison method (Nowell et al., 2017). Two themes emerged from the data: emphasizing the economic benefits of fracking, and minimizing environmental links to reproductive hardship. Both themes are discussed in detail below. Throughout this paper, all participants are referred to using pseudonyms.

## Results

Our analysis revealed that the women interviewed largely remained supportive of fracking due to its economic benefits while minimizing its potential links to their personal reproductive struggles. CDT Theory helps to explain our findings, as these themes indicate dissonance between cognitions about the economic benefits of fracking, potential harmful links between fracking and reproductive health, and a community identity rooted in part in the oil and gas industry. Overall, interviewees reduced dissonance by downplaying potential links between fracking and their personal reproductive health, thus enabling the cognitive maintenance of fracking as beneficial and unrelated to their health challenges.

### Emphasizing the Economic Benefits of Fracking

Participants were quick to emphasize fracking as an economic necessity. Although many acknowledged that safety practices could be improved upon, nearly all participants conveyed strong support for fracking. Of all participants, Michelle, a social worker originally from outside the area, was the most leery of fracking safety. Yet even Michelle acknowledged that her community was unlikely to support stricter regulations, much less a total ban on the practice. She explained, “That’s where our income comes from. That’s where, that’s where [sic] the money to support community interests come from.” Along with emphasizing the economic necessities of fracking, participants simultaneously minimized environmental concerns, arguing that too much regulation could curb economic benefits.

Participants frequently spoke to the positive economic outcomes of fracking operations. For example, Rita, a stay-at-home mom whose husband works in the oil and gas industry, noted that fracking enabled the construction of a new jail, a community conference center, and other public buildings, all of which were multimillion-dollar projects. She emphasized fracking as “important. . . . to our infrastructure, as well as just our work life.” In addition, Claudia, a stay-at-home mom with no immediate economic links to the industry (her husband works in healthcare), emphasized fracking as bringing substantial work to the community by bolstering business for “mom and pop shops” and creating employment for those who could work directly on fracking operations. Lindsay, who works for the health department and whose husband works as a miner, affirmed the dangers of fracking yet discussed it as a “necessary evil.” Though many of the women interviewed noted the dangers fracking poses to the environment, human health, and/or workers who risk injury or death on the job, the majority described economic benefits as substantial and essential.

Rita and Lindsay invoked economic benefits as reason to dismiss or minimize environmental concerns, thus reducing cognitive dissonance. That is, although closure or even regulation of oil and gas operations could improve public health and safety, participants cognitively (and communicatively) constructed economic losses as simply too costly. As Rita noted, “[We] want to make things safe for all parties involved, but not to the point of regulating it so much that it can’t be done or used.” She thus argued that practices should be safe but only insofar as safety precautions did not infringe on the industry’s ability to frack and reap economic benefits. Rita elaborated, “[T]here comes a point where you can regulate something to death where you can no longer provide for your family. . . . if we regulate it to death, then you’re going to have a bunch of unemployed people.” Once again, the consequences of mitigating health and safety concerns were, for participants, too costly. Rita’s sentiments suggested that any concerns needed to be considered in light of economic benefits. Immediate economic livelihood took precedent over potential health and environmental impacts. Such constructions were necessary for participants to reduce dissonance, allowing them to view fracking as permissible even in the case of concerning health effects.

### Minimizing Environmental Links to Reproductive Hardship

As noted earlier, pregnant women and their fetuses are particularly vulnerable to the effects of toxic chemicals, including those used for fracking (Layne, 2001; Currie et al., 2017; Whitworth et al., 2018; McKenzie et al., 2019). Though all our participants experienced some form of reproductive hardship, only one, Michelle, suspected her struggles were related to her proximity to fracking. The majority of interviewees dismissed or minimized the possibility of links between fracking and reproductive struggles. Several invoked the fact that they were originally from elsewhere as evidence that their reproductive challenges were unrelated to local fracking. Further, participants tended to elevate genetic and other personal factors as more likely than fracking to have caused reproductive challenges. Once again, these tendencies reflect CDT’s claim that uncomfortable dissonance leads individuals to cognitively reduce incongruities between cognitions or between cognitions and behaviors.

Claudia and Rita suggested that the fact that they grew up elsewhere made it unlikely that fracking in the Uintah Basin contributed to their reproductive hardship. When asked whether her experiences with miscarriage had impacted her perception of the fracking industry, Claudia replied, “I haven’t seen an impact of fracking. I also was not born and raised here. . . . So for me, no.” Rita emphasized the fact that she was originally from another area as reason to eliminate local fracking as a cause of her reproductive struggles: “I’ve dealt with reproductive issues, but I don’t think that they have anything to do with fracking. I didn’t originate here in the basin, and I haven’t always been around fracking and natural gas and all that stuff.” Rita also believed studies conducted in the region—designed to assess potential links between fracking chemicals and health—were flawed in that the majority of study participants “didn't originate from here . . . [and] didn't necessarily even work in the industry.” Rita and Claudia perceived fracking as only likely to impact the health of those who grew up near operations or had direct ties to the industry.

Relatedly, numerous participants attributed their hardships to genetic and/or personal factors. After noting that she was not born and raised in the Uintah Basin, Rita stated, “So I believe that it probably has more of a … something to do with my genetics, and just my own personal health issues that I deal with rather than an environmental cause.” Rita further emphasized that she was considered at an advanced age during the pregnancy and also noted that her husband had been working nights, creating exhaustion. While such factors can certainly impact pregnancy, other participants echoed an emphasis on genetic factors as reason to dismiss potential environmental links to their difficulties. Angela, a nurse and mother of two, stated, “I would say in my case it’s probably more genetic,” and yet another participant suggested her miscarriage was more likely linked to genetic as opposed to environmental factors. Such perceptions confirm Layne’s (2001) finding that women tend to take personal responsibility for pregnancy loss even in cases wherein environmental factors have been clearly established as contributors to reproductive harm. While taking personal responsibility for reproductive hardship “places an inordinate moral burden on women” (Layne, 2001, p. 41), it also offers cognitive benefits to women who take such (unfair) responsibility. That is, it allows them to reduce cognitive dissonance about the possibility that such hardship might be caused by industries that support their communities and/or their own families.

### Limitations

Although the themes noted in these interviews shed light on how women living near fracking operations make sense of their reproductive hardships, this study has limitations that warrant review. Miscarriage, stillbirth, birth defects, and difficulty with conception are deeply personal and even stigmatized issues, making women who have experienced such problems a population that is relatively hard to reach. Our study is thus limited in that we acquired only six participants whose insights cannot be generalized but are nonetheless informative. Further, the Uintah Basin’s economic dependence on fracking creates stigma for anyone speaking out against potential health consequences of the industry, as evidenced in the death threats received by the local midwife (Solotaroff, 2015). It is thus possible that the women interviewed *did* perceive some sort of link between fracking and their reproductive struggles but were unwilling to voice their concerns.

## Conclusion

This study aimed to address the dearth of literature discussing women’s perceptions of reproductive hardship in relation to hydraulic fracturing operations. Through in-depth interviews, we sough to investigate how and to what extent women living in the Uintah Basin—an area economically dependent on hydraulic fracturing—perceived their reproductive hardships as related to local fracking operations. Our findings, explained through CDT, illustrate that those interviewed played up the economic benefits of fracking at the same time that they downplayed fracking as a potential contributor to their experiences with miscarriage and other reproductive challenges. Thus, it appears that women continue to bear personal responsibility for reproduction while echoing industry rhetoric that pits jobs against environmental welfare (Estabrook et al., 2007). While such beliefs and communication patterns benefit such women in that they enable the reduction of dissonance, there is ample cause for concern in regards to health consequences caused by fracking operations. We want to conclude this paper by noting that a healthy economy is one that is sustainable in the long-term (Murphy, 2020). Rather than relying upon a toxic and tentative “boom-or-bust” economy, the United States in particular must make the move toward economies that value the ways in which human health is inextricably linked to the health of the environment.

## Data Availability

The original contributions presented in the study are included in the article, further inquiries can be directed to the corresponding author.
